# Temporal dynamics of face adaptation

**DOI:** 10.1167/jov.22.11.14

**Published:** 2022-10-27

**Authors:** Yi Gao, Jarod Pieller, Michael A. Webster, Fang Jiang

**Affiliations:** 1 School of Psychology, Georgia Institute of Technology, Atlanta, GA, USA; 2 Department of Psychology and Graduate Program in Integrative Neuroscience, University of Nevada, Reno, NV, USA

**Keywords:** visual adaptation, face gender, temporal dynamics

## Abstract

The appearance of a face can be strongly affected by adaptation to faces seen previously. A number of studies have examined the time course of these aftereffects, but the integration time over which adaptation pools signals to control the adaptation state remains uncertain. Here we examined the effects of temporal frequency on face gender aftereffects induced by a pair of faces alternating between the two genders to assess when the aftereffects were pooled over successive faces versus driven by the last face seen. In the first experiment, we found that temporal frequencies between 0.25 and 2.00 Hz all failed to produce an aftereffect, suggesting a fairly long integration time. In the second experiment, we therefore probed slower alternation rates of 0.03 to 0.25 Hz. A rate of 0.0625 Hz (i.e., 8 seconds per face) was required to generate significant aftereffects from the last presented face and was consistent with an average time constant of 15 to 20 seconds for an exponentially decaying integration window. This integration time is substantially longer than found previously for analogous effects for alternating colors, and thus points to a potentially slower mechanism of adaptation for faces compared with chromatic adaptation.

## Introduction

Our visual system is required to process a wide range of information received from the vast number of objects we encounter every day. For example, the light intensity changes by orders of magnitude within a day. A system that encodes the entire range of the light levels would be inefficient. Visual adaptation allows the visual system to quickly adjust its response to encode a variant range of stimuli, while maintaining sensitivity to variations around the ambient adapting level (Barlow, 1961; Fairhall, Lewen, Bialek, & van Steveninck, 2001; Simoncelli, 2003). Visual adaptation has been reported for low-level visual features such as contrast and spatial frequency (Engel & Furmanski, 2001; Greenlee, Georgeson, & Magnussen 1991; Wilson & Humanski, 1993), as well as more complex stimuli such as faces (Leopold, Rhodes, Mueller, & Jeffery, 2005; Rhodes, Jeffery, Clifford, & Leopold, 2007; Webster, Kaping, Mizokami, & Duhamel, 2004). Moreover, previous studies have shown that the visual system can adapt to not only general configural properties of faces (Webster & MacLin, 1999; O'Leary & McMahon, 1991), but also to the natural configural and featural information conveying information about age, ethnicity, or expression (Ng, Ciaramitaro, Anstis, Boynton, & Fine, 2006; O'Neil & Webster, 2011; Russell & Fehr, 1987; Webster et al., 2004) or individual identity (Leopold, O'Toole, Vetter, & Blanz, 2001). These adaptation effects may play an important role in ongoing calibrations of face processing mechanisms (Webster & MacLeod, 2011); therefore, it is important to understand the timescales over which these calibrations occur.

Previous studies of face adaptation have examined the temporal dynamics of adaptation by measuring the build-up and decay of aftereffects induced by adapting to different durations of exposure. Studies using a single adapting stimulus have pointed to both similar dynamics for faces and contrast adaptation (e.g., Leopold et al., 2005; Rhodes et al., 2007) and some long-lasting components of face adaptation (e.g., Carbon et al., 2007; Carbon & Ditye, 2011; reviewed in Strobach & Carbon, 2013). In this study we instead examined the temporal properties of adaptation to an alternating pair of faces, following the logic of experiments previously applied to examine the time constants of adaptation for color perception (Webster & Wilson, 2000). In their study, participants matched the color of a gray test field after adapting to fields whose color varied sinusoidally at different temporal frequencies. The mean chromaticity of the flicker equaled the gray background. If the time constant for the adaptation is slow relative to the flicker rate (e.g., so that the adaptation level was based on the signal integrated over multiple cycles of the flicker), then the flickering stimuli should produce little or no net color aftereffect. Conversely, if the adaptation has a short time constant relative to the flicker rate (e.g., so that adaptation integrated only over a portion of a cycle) then the color aftereffect should be largely determined by the last color shown, and the aftereffects should depend on the phase of the flicker. We used a similar procedure to examine gender aftereffects^1^ by adapting to alternations between a male and female face at different rates. The rates at which different face aftereffects emerge for a different phase of the alternation (male face last vs. female face last) can, like chromatic adaptation, help to reveal the temporal window over which adaptation to faces occurs.

## General methods

### Observers

Ten participants were recruited for Experiment 1 (five male and five female; aged 19–27 years old; mean, 22.7 ± 2.3 years). Another 10 participants were recruited for Experiment 2 (four male and six female; aged 19–34 years old; mean, 24.0 ± 5.4 years). All participants were students at the University of Nevada, Reno. All had normal or corrected-to-normal vision and participated with informed consent. Study protocols were approved by the Institutional Review Board of University of Nevada, Reno.

### Stimuli

All stimuli were presented on a calibrated Display++ LCD monitor (Cambridge Research Systems, Rochester, UK). The monitor was refreshed at 120 Hz. The adapting faces were one female and one male face, averaged from eight female (AF01, AF06, AF09, AF11, AF13, AF29, AF34) and eight male faces (AM02, AM03, AM05, AM06, AM07, AM11, AM29, AM31) from the Karolinska Directed Emotional Faces database (Lundqvist, Flykt, & Öhman, 1998). For the test faces, 101 faces were morphed between the average female and male faces. A morphing level of 0 represented the average female face and a morphing level of 100 represented the average male face, with the 51st face (morphing level of 50) as a nominal neutral face. Faces from the Karolinska Directed Emotional Faces database were first converted to grayscale and then averaged and morphed using FantaMorph (Abrosoft, 2002–2020). External features like hair or ears were cropped. All adapting faces subtended a width of 4° and a height of 5°. All test faces subtended a width of 3.40° and a height of 4.25°, with the size difference included to decrease the impact of low-level (e.g., light adaptation) aftereffects (Zhao & Chubb, 2001). All faces were presented in the center of the visual field and participants sat 75 cm from the display and viewed the stimuli binocularly in an otherwise dark room.

For both experiments, participants completed 2 to 10 training sessions before the actual experiment (depending on individual performance) to ensure they could reliably discriminate the gender of faces before testing. Because we had 101 faces morphed between the average female and male faces for the test stimuli, the changes between different morphing levels were subtle. Moreover, the faces were in grayscale and had a relatively small size (a width of 3.4° and a height of 4.25°). Therefore, practice training sessions were provided for participants to reach a criterion level of performance. All participants reached the criterion after 2 to 10 training sessions (50 trials for each training session).

## Experiment 1

### Procedure

**Figure 1.**
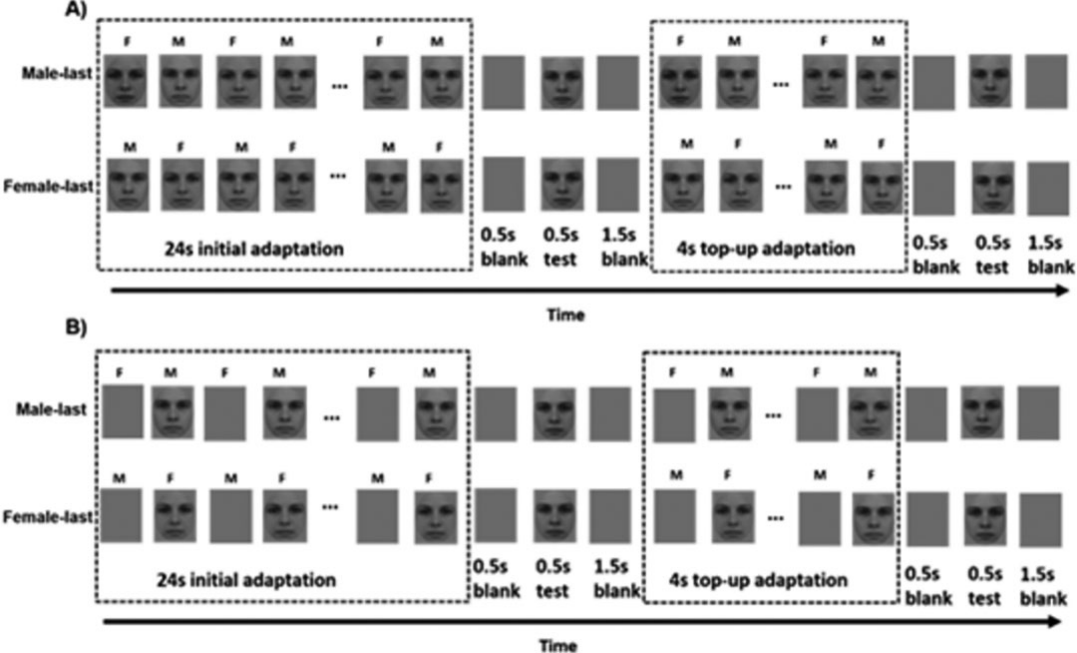
Example sessions of face-alternating condition (A) and control condition (B) in Experiment 1.

Each adaptation session started with a 24-second initial adaptation period and was followed by a 0.5-second blank screen. The test face was then presented for 0.5 seconds, followed by a 1.5-second blank screen and a 4-second top-up adaptation (see Figure 1 for experimental setups). On each trial, participants used a keyboard to indicate whether the test face appeared female or male. This top-up adaptation procedure was repeated after each trial until 50 trials were completed. Throughout the entire experiment, a red fixation dot (0.25° diameter) was presented at the center of the screen, and participants were asked to maintain their fixation on the dot.

Two conditions were included. In the face-alternating condition, male and female adapting faces were alternatingly presented during each top-up period. In the control condition, only one adapting face (male or female) was presented during the top-up period, alternating with a gray screen with a fixation. Four temporal frequencies were included to modulate the alternation rate between the two adapting faces (face-alternating condition) or between the adapting face and the gray screen (control condition): 0.25 Hz, 0.50 Hz, 1.00 Hz, and 2.00 Hz (corresponding with 2 seconds, 1 second, 500 ms, and 250 ms per face adaptor/gray screen). In addition, two face presentation orders were tested, so that each top-up ended with the female adapting face (female last) or the male adapting face (male last) (Figure 1).

A one-down one-up staircase with a fixed length of 50 trials was used to measure the adaptation effect in each session. The initial step size was three morphing levels and after three reversals it was decreased to two morphing levels. After another three reversals, the step size was fixed to one morphing level. The average of the last six reversals of the two staircases was calculated and used as the point of subjective equality (PSE). Each participant completed a total of 16 sessions (2 conditions x 4 temporal frequencies x 2 face presentation orders). Sessions were tested on different days, with one-half the participants completing the eight face-alternating sessions first and the other one-half completing the eight control sessions first. The testing order of eight experimental and control sessions was randomized.

We also measured the PSE without adaptation. For the session without adaptation, each trial started with a 0.5-second test face, followed by a 1.5-second gray screen with fixation dot only, during which participants were asked to judge whether the test face appeared female or male. The number of trials and the method to calculate the PSE was the same as in the adaptation sessions. Participants’ natural bias for the gender category boundary was defined by the difference between the PSE without adaptation and the nominal neutral face level (50). The aftereffect was calculated by subtracting the bias from the PSE measured after adaptation and is plotted relative to the nominal neutral point of 50.

### Results

**Figure 2.**
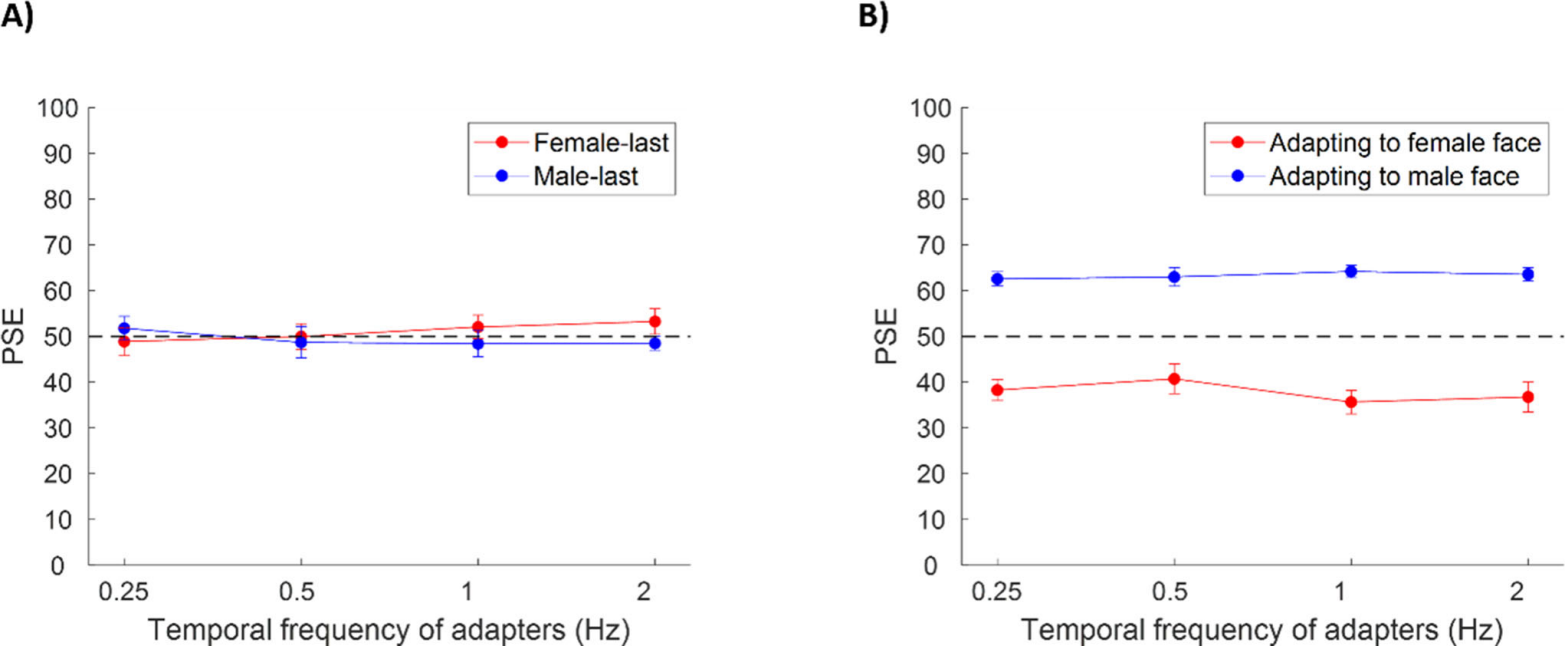
Average of PSEs (after accounting for bias) across 10 participants for both the face-alternating condition (A) and the control condition (B). The dashed line corresponds to no shift in the PSE (i.e., no aftereffect). The error bars represented 1 standard error.

The left panel of Figure 2 shows the PSEs for the face-alternating condition and the right panel of Figure 2 shows PSEs for the control condition (after accounting for bias). For the typical face gender aftereffect, a neutral test face would look more like a male face after adapting to a female face. In this case, aftereffects would be revealed by the PSE shifting toward the female face, because this stimulus bias is necessary to null out or cancel the perceptual bias induced by the adaptation. Similarly, the aftereffects of adapting to a male face would be revealed by PSE shifts toward the male face. We ran a 2 (experimental condition: face-alternating vs. control) × 4 (temporal frequencies: 0.25 Hz, 0.50 Hz, 1.00 Hz, and 2.00 Hz) × 2 (face presentation order: male last vs. female last) repeated measures analysis of variance using JASP (JASP Version 0.14.1, Computer software) on the PSEs. The interaction among face presentation order, temporal frequency, and experimental condition was not significant, F (3, 27) = 1.0, *p* = 0.4. The interaction between experimental condition and face presentation order was significant, F (1, 27) = 55.6, *p* < 0.001. A simple main effect analysis showed that, in the control condition, the PSEs of male-last condition (mean, 63.3 ± 5.0) were significantly larger than those of female-last condition at all temporal frequencies tested (mean, 37.8 ± 8.9), paired *t* test with Bonferroni correction, *t* (39) = 17.5, *p* < 0.001, indicating a typical face gender aftereffect. In the face-alternating condition, no significant difference was revealed between female-last (mean, 51.0 ± 8.7) and male-last (mean, 49.3 ± 8.3) condition, paired *t* test with Bonferroni correction, *t* (39) = 1.2, *p* = 1.

To summarize, we found that for temporal frequencies ranging from 0.25 to 2.00 Hz, alternating a pair of faces of different genders effectively canceled gender aftereffects at all temporal frequencies tested. Moreover, when adapting to a single face (alternated with gray screen) for a constant total duration, the magnitude of aftereffects did not depend on the temporal frequency of face presentation. Thus, this finding suggests that the integration time controlling face adaptation was substantially longer than the time allowed by the range of frequencies tested. In Experiment 2, we therefore probed the aftereffects at a range of slower temporal frequencies.

## Experiment 2

### Procedure

Ten additional participants were recruited for Experiment 2. Because of the much longer adapting duration required for the lower temporal frequencies, we changed the staircase to a trial-by-trial design with a rating task and increased the adaptation duration in each trial to 32 seconds. This practice allowed us to assess the aftereffects after a single adapting period rather than sequentially with multiple top-ups as in Experiment 1. The temporal frequency of the face alteration during the adaptation period was again manipulated across runs. Four temporal frequencies were tested: 0.03125 Hz, 0.06250 Hz, 0.12500 Hz, and 0.25000 Hz (corresponding to 16, 8, 4, or 2 seconds per face adaptor). Each trial started with a 32-second adaptation period and was followed by a 0.5-second blank screen and a 0.5-second neutral test face. Participants were then given a 3.5-second response time, during which they were asked to use the mouse to slide on a scale from 1 to 7 to indicate the gender of the test face, with 1 being female, 7 being male, and 4 being neutral. Similar to Experiment 1, two face presentation orders were tested, so that the adaptation period ended with the female adapting face (female last) or the male adapting face (male last). Each condition was repeated once per block and there were seven blocks in each session. Two sessions were run for each participant. Therefore, there were 14 ratings for each condition.

To measure the bias of rating of the neutral face without adaptation, each trial started with a 0.5-second test face after which the observer was given 3.5 seconds to rate the gender. There were 10 ratings for the neutral face. The bias was defined by the difference between the PSE without adaptation and the objective neutral face level (4). The aftereffect was calculated by subtracting the bias from the ratings measured after adaptation.

Although our measurements do not reveal the shape of the integration window for adaptation, we estimated the time constants by fitting a simple exponential decay model. This was used to describe the adaptation effect at time *t*,

$$\begin{array}{c} f\left(t\right)=A_{1}*\left(e^{-\frac{t}{tau}}\right),\; \end{array}$$

where *A*_1_ represented the stimulus history, and *tau* represented the time constant of the decay function. The measured aftereffect in the current experiment was the sum of the aftereffects at each time point *t* and was calculated using the following formula:

$$\begin{array}{c} Y=\sum_{i=1}^{t}f\left(t\right),\; \end{array}$$

with the total aftereffect normalized by the total area under the curve. The normalized aftereffect with a bias term was then fit to the data for each condition to estimate the time constant for the adaptation.

### Results

**Figure 3.**
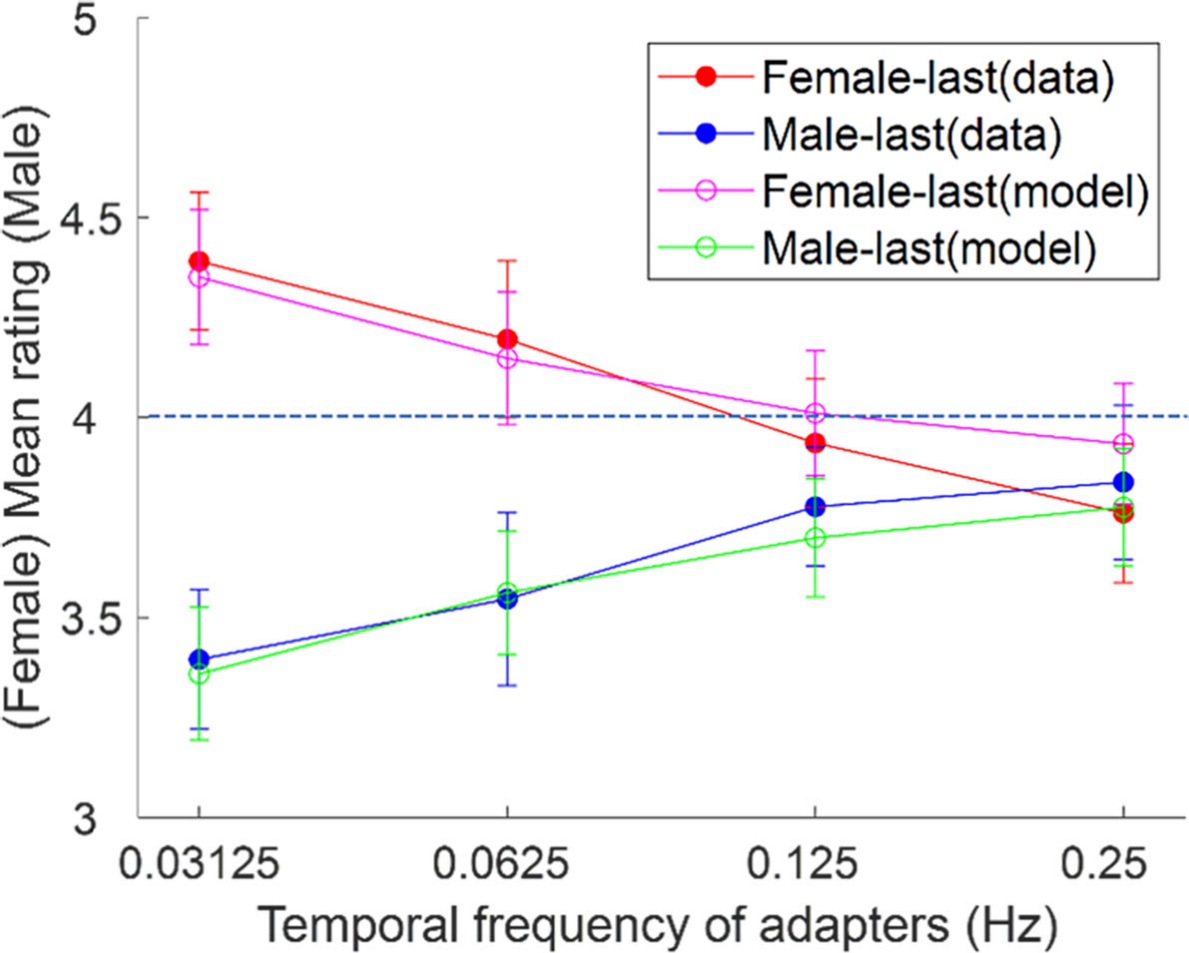
Ratings of the gender of the test face (after accounting for bias) in Experiment 2 when the adaptation period ended with female adaptors (solid red circles) or male adaptors (solid blue circles). The lines with unfilled circles represent estimates based on an exponential decay model. The error bars represented 1 standard error. The dashed line indicated there was no aftereffect.

Figure 3 showed the results of Experiment 2, with the observed settings shown by the red and blue symbols. We ran a two-way repeated measures analysis of variance to compare the mean ratings after adaptation (after accounting for bias) as a function of temporal frequency (0.03125 Hz, 0.06250 Hz, 0.12500 Hz, and 0.25000 Hz) and face presentation order (female last and male last). The interaction between the two independent variables was significant, F (3, 27) = 6.8, *p* = 0.002. A post hoc analysis with Bonferroni correction showed a significant difference between the two face presentation orders at 0.03125 Hz, *t* (9) = 5.3 *p* < 0.001; female face last, mean, 4.3 ± 0.6; male face last, mean, 3.3 ± 0.5; 0.06250 Hz, *t* (9) = 4.9, *p* < 0.001, female face last, mean, 4.2 ± 0.7, male face last, mean, 3.5 ± 0.7. However, differences were no significant for the 0.12500-Hz and 0.25000-Hz alternation rates (all *p*s = 1) with Bonferroni correction. The results were thus consistent with Experiment 1 in revealing no phase-specific adaptation effects at 0.2500 Hz, although these effects did emerge for frequencies of 0.0625 or lower. Fits of the exponential decay model to the data provided an estimate of the average time constant for the decay of 19.5 ± 13.6 seconds.

## Discussion

The current study examined the effects of face gender adaptation to a pair of alternating female and male faces. We found that when faces of different genders were alternated slowly (longer than 4 seconds per face), the aftereffects were more strongly determined by the last face of the adaptors. When faces were alternated at a faster rate (4 seconds per face or shorter), the gender aftereffects induced by a pair of alternating female and male faces were canceled out and were determined by the mean of the adapting faces. The average aftereffects were well predicted by an exponential decay function with a time constant of approximately 20 seconds.

Our results for gender adaptation are broadly consistent with a previous unpublished study of face aftereffects using a similar paradigm but tested with facial distortions (Muskat, 2001). Muskat (2001) found that phase-dependent face aftereffects were emerging by 0.250 Hz and become stronger by 0.125 Hz, suggesting a somewhat more sluggish mechanism than what Webster and Wilson (2000) observed for color aftereffects. However, we found no evidence for phase-specific face aftereffects at these frequencies in the current study. This difference is further pronounced because color flicker also induces strong contrast adaptation (to the stimulus variation) in addition to chromatic adaptation (to the stimulus mean) (Webster, 1996). This contrast adaptation further suppresses the afterimage induced by the mean adapting level, suggesting that the response driven by the mean was likely to be faster. Analogous adaptations to facial contrasts have been found, but by comparison are substantially weaker than for chromatic contrast (Gwinn, Retter, O'Neil, & Webster, 2021; Muskat, 2001). The timescales we found are also roughly consistent with neural measures of adaptation to faces. Mattar, Kahn, Thompson-Schill, and Aguirre (2016) used functional magnetic resonance imaging to measure brain responses during the presentation of a series of synthetic faces varying in identity, skin tone, and gender simultaneously. They showed that the neural responses to the current face were modulated by the average stimulus history, as predicted by an exponential integrator for the influence of previous faces. The half-life of this exponential decay of the neural responses in the fusiform face area was 7.5 seconds (the average time constant of decay was 19.5 seconds in our experiment, corresponding to a half-life of 13.5 seconds). Taken together, this finding suggests a longer timescale for face adaptation than chromatic adaptation.

The sites of chromatic adaptation are primarily retinal (Zaidi, Ennis, Cao, & Lee, 2012). Previous studies of face adaptation dynamics have instead pointed to similar timescales compared with cortical contrast adaptation, even though these studies have tried to isolate higher level attributes of faces (Rhodes et al., 2007). The dynamics of adaptation are likely to be tuned to ecologically important timescales of variation in the world and to the task demands of the observer. For example, chromatic adaptation needs to be slow enough to integrate over multiple fixations (e.g., to adapt to the average illumination) and fast enough to adjust to the large local changes in mean luminance and contrast at different locations within a scene (Rieke & Rudd, 2009). In general, temporal processing and integration appear to slow at higher stages of the visual pathway. For example, Mattar et al. (2016) found that the temporal integration timescale increased along the visual hierarchy (V1–V2–V3–fusiform face area), with the half-life increasing from 0.8 to 7.5 seconds. In the macaque cortex, the temporal receptive window for visual input increases along the visual processing hierarchy (Chaudhuri, Knoblauch, Gariel, Kennedy, & Wang, 2015). Thus, our results, compared with Webster and Wilson (2000)’s findings for retinal chromatic adaptation, are consistent with neuroimaging and animal studies that the timescale of visual integration broadens along the visual hierarchy.

However, it is also clear that adaptation for a given trait can occur over multiple timescales (Kording, Tenenbaum, & Shadmehr, 2007). It can be induced after a few milliseconds of observation (Glasser, Tsui, Pack, & Tadin, 2011; Priebe, Churchland, & Lisberger, 2002) and can persist as long as days to even months or years (Belmore & Shevell, 2011; Dodwell & Humphrey, 1990; Haak, Fast, Bao, Lee, & Engel, 2014; Robinson & MacLeod, 2011). For example, color aftereffects after cataract surgery last for months (Delahunt, Webster, Ma, & Werner, 2004). Distinct short- and long-term timescales have also been demonstrated for face adaptation (Mesik, Bao, & Engel, 2013; Carbon & Ditye, 2011). It is an open question as to what ecological or mechanistic demands might have shaped the rates of adaptation for different visual attributes.

Our results also have practical implications for designing face adaptation experiments. Often studies of adaptation vary the adapting stimulus (e.g., jittering the position or properties to control for low-level confounds like light adaptation) (Leopold et al., 2001; Moradi, Koch, & Shimojo, 2005; Rhodes et al., 2007). Studies of face adaptation have also used collections of faces to examine how observers adapt to the average property (Ying & Xu, 2017) or to the common traits of the set (Webster et al., 2004). Our results place a limit on the timescales over which this averaging might occur (for the short-term conditions we tested).

Our result of the control condition in Experiment 1 showed that when the total adaptation duration was the same (2 seconds), the adaptation effect to repeat presentation of a constant face (alternated with a uniform gray screen) was the same regardless of the temporal frequency of the face presentation. These results differ from the aftereffects for some visual properties such as numerosity. Aagten-Murphy and Burr (2016) showed that it was the number of adapting events but not the duration of each adapting event or the total adaptation duration that determined the overall adaptation magnitude. These differences point to the possibility that the dynamics of adaptation also depend on how and where different stimulus properties are processed. Although faces are processed primarily in the fusiform face area, numerosity perception is believed to be strongly dependent on higher regions of the cortex, such as the intraparietal sulcus (Dehaene, Piazza, Pinel, & Cohen, 2003; Piazza & Izard, 2009). Future studies are needed to evaluate the aftereffects of different stimulus types by comparing timescales of adaptation across multiple stimulus dimensions.

## Footnotes

1 Note that we use the term “gender” rather than “sex” throughout the manuscript as we aimed to study how adaptation changes people's perception and interpretation of whether a person looks more like a female or male. The term “sex” focuses more on the biological characteristics of the individuals. The usage of “gender adaptation” follows previous studies on gender adaptation (Barrett & O'Toole, 2009; Yang et al., 2011; Zhao et al., 2011).
